# Capturing drug use patterns at a glance: An *n*-ary word sufficient statistic for repeated univariate categorical values

**DOI:** 10.1371/journal.pone.0291248

**Published:** 2023-09-08

**Authors:** Gabriel J. Odom, Laura Brandt, Clinton Castro, Sean X. Luo, Daniel J. Feaster, Raymond R. Balise, the CTN-0094 Team

**Affiliations:** 1 Department of Biostatistics, Florida International University, Miami, FL, United States of America; 2 Department of Psychology, The City College of New York, New York City, NY, United States of America; 3 The Information School, University of Wisconsin-Madison, Madison, WI, United States of America; 4 Department of Psychiatry, Columbia University, New York City, NY, United States of America; 5 Division of Biostatistics, Department of Public Health Sciences, The University of Miami, Miami, FL, United States of America; Max Planck Institute for Solid State Research, GERMANY

## Abstract

**Introduction:**

The efficacy of treatments for substance use disorders (SUD) is tested in clinical trials in which participants typically provide urine samples to detect whether the person has used certain substances via urine drug screenings (UDS). UDS data form the foundation of treatment outcome assessment in the vast majority of SUD clinical trials. However, existing methods to calculate treatment outcomes are not standardized, impeding comparability between studies and prohibiting reproducibility of results.

**Methods:**

We extended the concept of a binary UDS variable to multiple categories: “**+**” [positive for substance(s) of interest], “**–**” [negative for substance(s)], “**o**” [patient failed to provide sample], “*” [inconclusive or mixed results], and “_” [no specimens required per study design]. This construct can be used to create a standardized and sufficient representation of UDS datastreams and sufficiently collapses longitudinal records into a single, compact “word”, which preserves all information contained in the original data.

**Results:**

We developed the R software package CTNote (available on CRAN) as a tool to enable computers to parse these “words”. The software package contains five groups of routines: detect a substance use pattern, account for a specific trial protocol, handle missing UDS data, measure the longest period of consecutive behavior, and count substance use events. Executing permutations of these routines result in algorithms which can define SUD clinical trial endpoints. As examples, we provide three algorithms to define primary endpoints from seminal SUD clinical trials.

**Discussion:**

Representing substance use patterns as a “word” allows researchers and clinicians an “at a glance” assessment of participants’ responses to treatment over time. Further, machine readable use pattern summaries are a standardized method to calculate treatment outcomes and are therefore useful to all future SUD clinical trials. We discuss some caveats when applying this data summarization technique in practice and areas of future study.

## Introduction

### Background

The onset of the COVID-19 pandemic coincided with millions of Americans reporting increases in their substance use “a little more or much more” [1], and over 40 million Americans were classified as needing treatment for a substance use disorder (SUD) in 2020 [2]. SUDs continue to be a major public health challenge, with drug overdoses in the United States topping 100,000 in 2021 [3]. SUDs are treated with a combination of pharmacological and psychological interventions, known as medication-assisted treatment [4].

The efficacy of these treatments is tested in clinical trials in which participants typically provide urine samples, to detect whether the person has used certain substances via urine drug screenings (UDS). UDS is a point-of-care test that gives a binary, qualitative readout of the presence of an array of illicit drugs and prescription medications. Similar longitudinal data of drug use can also be obtained through patient self-report, using a standardized schema called Time-line Followback (TLFB). For a patient in opioid use disorder treatment, for example, a test negative for opioids would typically be indicative of a positive outcome–the patient has not used opioids recently (note that the length of time a drug can be detected in the urine varies due to several factors, including dosing, duration of use, and the drug’s particular pharmacokinetics). In clinical practice and most clinical trials in SUD, treatment success is primarily determined by demonstrated improvement in patterns of UDS, and relatedly, TLFB. Proper presentation of these data streams is a ubiquitous challenge in SUD research.

For a single substance or group of substances, outcomes of these assessments are uni-dimensional and categorical. Common UDS categories include “substance positive”, “substance negative”, “improper urine sample temperature” (temperature readings are often used to detect adulterated/tempered urine samples), “missing urine” (usually indicative of a patient not showing up for their scheduled study visit), and others. Further, because urine is sampled sequentially over the course of a clinical trial, UDS are correlated within subject and across study weeks. Because of these properties, single-value summaries of the UDS pattern (such as the proportion of “substance positive” results, or the maximum number of consecutive “substance negative” results) are not statistically *sufficient* statistics. They do not contain all of the information about each trial participants’ UDS pattern that was contained in the original UDS data [5].

Importantly, UDS data form the foundation of treatment outcome assessment in the vast majority of SUD clinical trials. For example, a certain proportion of opioid-negative urine samples provided by a patient over the course of a treatment period is taken as evidence that the patient has ceased their regular opioid use and the treatment is working. However, existing methods to calculate treatment outcome are not standardized, requiring a complex code base that is difficult to reuse for investigators in the same field. These difficulties impede reproducibility of results or comparisons between studies—even if similar trial designs are used. A standardized data format for this particular data stream would have broad applicability in both clinical trials and other related activities, such as quality improvement research.

### Overview of this paper

In this paper, we extend the concept of a binary UDS variable to multiple categories and show that this can be used to create a standardized and sufficient representation of UDS datastreams. We show that this construct sufficiently collapses longitudinal records into a single, compact “word”, which preserves the information in the original data. We also present a few example algorithms to calculate some common single-value summaries from such a “word”. Finally, we introduce a standard and code-based library of algorithms (contained in the CTNote package [6] for the R programming language [7] and available on CRAN at https://CRAN.R-project.org/package=CTNote) for treatment outcome definitions useful to evaluate medication-based treatments for SUDs.

## Methods

### “Words” as sufficient statistics for longitudinal categorical data

We aim to create a sequence of letters and symbols, which are easy to interpret and remember, that act as a sufficient statistic for a categorical random variable observed in a longitudinal manner. Our motivating example is a compact and sufficient representation of a patient’s weekly UDS results. For a dipstick test which detects a substance of interest in the urine, this statistic is a representation of an individual participant’s full pattern of substance use. To be of use, this summary statistic must have the following properties:

1. (*Machine-Readable*) It can be directly parsed by a computer [8].
2. (*Human-Readable*) It can be quickly and easily interpreted by a human.
3. (*Sufficient*) It represents all of the same information about the sequence of interest that would be present in the full data [5].

### Binary and *n*-ary “words”

Binary words are a compact representation of a sequence of logical or binary variables [9]. For example, let a random variable named *x* indicate whether or not a clinical trial participant visited their clinic in each week over a four-week period, and assume that we observe the pattern: “visit”, “visit”, “no visit”, “visit”. If “1” represents a visit and “0” represents no recorded visit, then we can represent this clinic visit pattern as the binary word “1101”. Similarly, we could represent these two categories symbolically as “V” for visit and “_” for no visit, so our binary word becomes “VV_V”. Notice that for a short sequence like our 4-week example here, the original data itself is both machine readable and human readable. However, human readability begins to suffer as sequences grow longer—we can no longer grasp the whole pattern at a glance.

Extensions of this concept have been famously applied to the human genome [10]. The four nitrogen bases in DNA are abbreviated by their first letter: A for adenine, T for thymine, G for guanine, and C for cytosine. These four letters are collapsed by their position in the genome into a *quaternary* word (or a *quinary* word if U is included for uracil). For example, the “word” AAACCATTCACAATCAGACA expresses a sequence of 20 nucleic acid bases without loss of information about the bases (the “word” meets the definition of statistically *sufficient*). The “word” AAACCATTCACAATCAGACA is also much easier to understand by a human than the condensed structural formula or a skeletal-structural formula of these 20 compounds (i.e., the “word” is *human readable*). Furthermore, such a quaternary word can be parsed by a computer with ease (i.e., the “word” is *machine readable*).

### Quinary UDS pattern legend and presentation

We now build from the examples above. To compactly summarize a participant’s pattern of substance use (for a particular class of substances) over time, we first define the following five-value legend:

- +: positive for the substance(s) by urine screen (or participant self-report/TLFB, if such data are of interest) at a specific visit or in a specified window of time (a day, week, etc.)
**−**: negative for the substance(s)
- **o**: patient failed to provide a urine sample
*: inconclusive results or mixed results (e.g., patient provided more than one urine sample in the time interval and the results did not agree)
- _: no specimens required per study design (weekends, holidays, pre-randomization period, alternating visit days/weeks)

We note that the “+” and “-” symbols have been used in clinical shorthand (e.g., in patient files) for decades. Obviously, this legend can be modified and extended to better reflect the complexities of individual clinical trials or practice settings, but we believe that the structure (representing visits / short time intervals as single symbols) will still be beneficial as a “snapshot” of the patient’s treatment results and adherence to trial protocol, and also as a computable summary of the patient’s behavior during the follow-up period and post-trial analysis phase.

### A use pattern example

The following example (Table 1) represents a real, deidentified participant in a clinical trial of medication-assisted treatment for opioid use disorder. We will first observe the recorded opioid use data via urine screen for this participant after randomization. The variable who indicates the participant ID, result refers to the result of the urine drug screen (Positive, Negative, or Missing [i.e., patient failed to provide sample]) for the substance of interest (opioids only for this trivial example), and when shows the study day on which this observation was made.

**Table 1:** Opioid use pattern for an example participant; “…” represents a series of UDS results negative for opioids.

who	result	when
2,089	Positive	0
2,089	Positive	7
2,089	Positive	15
2,089	Positive	21
2,089	Positive	28
2,089	Negative	35
	…	
2,089	Positive	56
2,089	Negative	63
	…	
2,089	Missing	162
2,089	Negative	169

Once data are in this form, data can be parsed by a computer (*machine readability*). Additionally, this table represents all available opioid use information for this participant (*sufficiency*). However, data in this form are **not easy to interpret** directly by a researcher or clinician (not *human readable*). As can be seen in this table, it is somewhat difficult to visualize this patient’s treatment trajectory using data in this form, but it is possible to display here because there are two long periods of complete opioid abstinence (“Negative”) which can be summarized with an ellipse (…) symbol. Data for a more complex patient, with sporadic opioid use (opioid-positive UDS) or abstinence (opioid-negative UDS), would require several pages to print. Using this style of long-form data prevents deriving clinical intuitions at a glance about an individual patient or the efficacy of the treatment overall.

In contrast, observe the opioid use pattern summary for this same participant:

+++++---+--------------o-.

Using the basic pattern definition legend above, we can clearly see that this patient was positive at baseline (+), had a challenging first month (++++), improved in the second month with only a single lapse (---+), and then remained abstinent from opioids for the remainder of the clinical trial with a single missed visit towards the end of the treatment period (--------------o-). Participant substance use pattern data in this form are **easy to interpret** clinically. Moreover, this symbolic representation of substance use can be parsed and summarized by a computer. Finally, notice that this pattern represents the entire course of treatment for this patient without loss of any clinically relevant information about their opioid use as objectively defined by UDS results.

Use pattern words should, if possible, be displayed in fixed width/monospace fonts. That is, each character should use exactly the same space on the page. If such a font is used and several “words” are displayed left-aligned on a page, it is easy for a human to scan up and down a “column” of symbols to see what was observed for every patient on any visit (e.g., what happened on the fifth visit).

**Fig 1:**
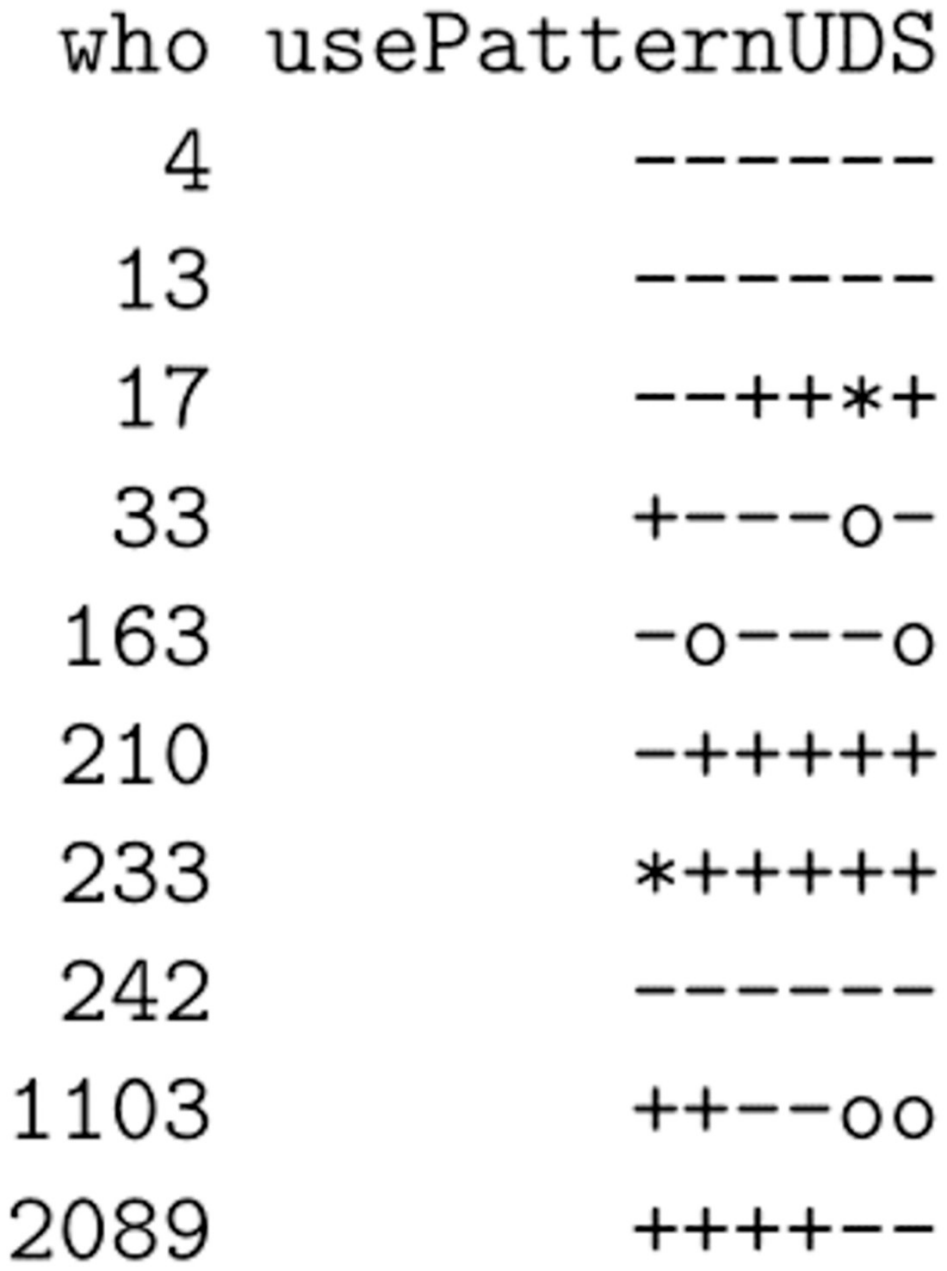
Short-term use patterns as “words” for multiple patients.

One last major strength is that using these opioid summary words allows for quick inspection of multiple patients’ treatment trajectories (and clusters) all at once. Instead of checking multiple charts one-by-one to get a rough assessment of the treatment efficacy, we can quickly see overall study arm patterns at a glance. The output shown in Fig 1 is an example of what these urine screen “words” would look like after patients had completed 6 weeks of a study post baseline. This gives us a quick visual summary of which participants have responded well to the treatment so far (4, 13, 33, 163, and 242) and which participants have not (17, 210, 233, 1103, and 2089). Such quick clinical insights would be challenging to achieve from the raw data alone.

## Results

Besides being easy to interpret clinically, these use pattern summaries are also “machine readable” (a computer can parse them). Therefore, they not only aid clinical interpretations but they may also serve as a standardized method to calculate treatment outcome. An add-on software package for the R programming language, called CTNote, provides functions to process these use patterns [6]. The table (Table 2) below shows the functions grouped by type provided in this package.

**Table 2:** Functions in the package CTNote, their categories, and their descriptions.

Function Name	Description
**Count Substance Use Events**	
count_matches()	Count Periods of Substance Use / Abstinence
**Detect a Substance Use Pattern**	
detect_subpattern()	Detect a Consecutive Sub-Pattern
detect_in_window()	Detect Visits before Matching a Fuzzy Sub-Pattern
**Measure the Longest Periods of Consecutive Behavior**	
measure_retention()	Measure Length of Use Pattern before Dropout
measure_abstinence_period()	Measure the Length of the Longest Abstinent Period
**Handle Missing UDS Data**	
recode_missing_visits()	Recode Missing or Ambiguous UDS in a Subject Use Pattern
impute_missing_visits()	Naively Impute Missing Visits
**Account for the Study Observation Design or Trial Visit Protocol**	
collapse_lattice()	Combine Multiple Simple Study Design “Lattices”
view_by_lattice()	View a Pattern through a Study Design “Lattice”
**Other Functions**	
weight_positive_visits()	Weight Visits in a Subject Use Pattern ()

By executing various permutations of these routines, we were able to write algorithms to define an array of treatment outcome definitions commonly used in SUD clinical trials. In our supplemental material, we include a “library” of algorithms to calculate each of these definitions (also available under “Endpoints Library” on the CTNote website: https://ctn-0094.github.io/CTNote/). The input of each algorithm is a set of substance use pattern summaries (as a “word”) for all clinical trial participants; the output of each algorithm is a calculated treatment endpoint for each participant included.

Below, we provide three examples representing primary endpoints in seminal clinical trials of opioid use disorder treatment. The CTNote package was designed to follow principles outlined in the Tidyverse Style Guide to produce reader-friendly code [11]. To understand the code, be aware that R uses the symbol # to indicate that the rest of a line is an explanation for the reader, and the symbol |> means do this task “and then” send the results to the next task/function. In R, one or more details for functions are provided inside the function’s parentheses. As you will see in later examples, if a function needs many details they can be listed as a comma delimited list inside of the parenthesis.

### Example: Longest period of abstinence

One of the treatment endpoint definitions used in [12] is “the maximum consecutive days abstinent” from opioids, where missing clinic visits are imputed to represent a urine screen positive for the substance of interest (here: opioids). The algorithm to calculate this definition for our example patient is written in R code, shown below:

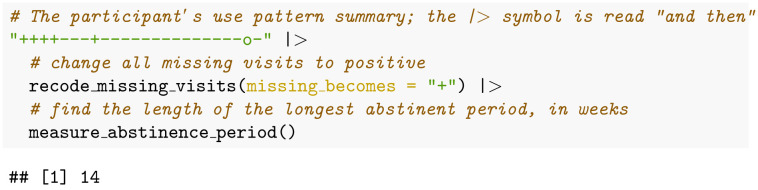


This code produces the value 14, the number of consecutive weeks during which the patient did not use opioids. This code is easy to write, and the code is transparent to understand–even by an R coding novice.

### Example: Percentage abstinence after grace period

The primary efficacy endpoint definition used by [13] is the “percentage of opioid-negative UDS from week 5 to week 24”. Therefore, the code to calculate this definition for our example patient would be:

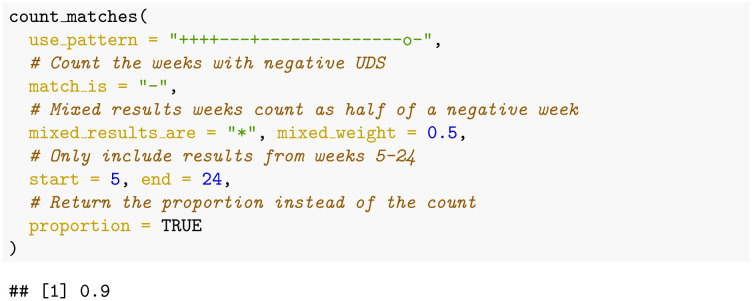


This code returns the value 0.9 (90%), which is the proportion of weeks wherein the patient did not use opioids. While the count_matches() function requires the author to provide many details, we believe that this function’s detailed “arguments” are well named and they make the code easy to understand.

### Example: Relapse to substance use

The primary endpoint “relapse” used by [14] was defined as “three consecutive opioid-positive urine tests”, where missing clinic visits are imputed to represent a urine screen positive for the substance of interest (again, opioids here). Therefore, the algorithm to calculate this definition for our example patient would be:

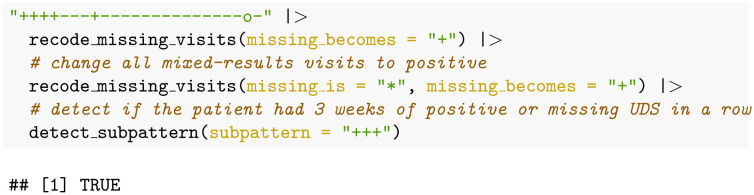


This code returns the R key word “TRUE” meaning that the participant did have three consecutive urine tests positive for opioids.

If we were interested in relapse from a “time-to-event” or reliability perspective [15], the algorithm above changes only in the last function call:

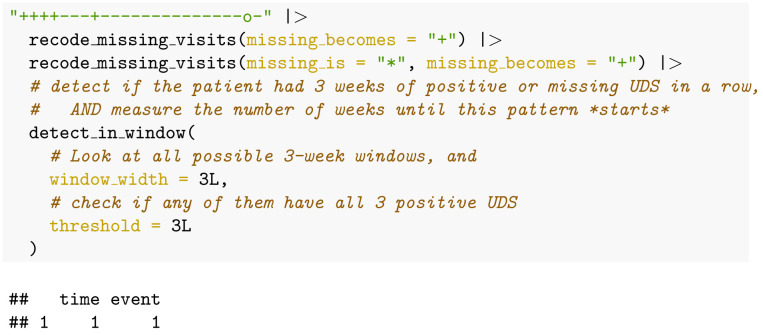


This code returns a data table with two columns/variables. The first column, named “time”, holds “1”: the first week in the observed pattern of 3 or more weeks of consecutive use was week 1. The second column, named “event”, holds the value “1”, indicating the pattern was observed (vs. a value of 0 otherwise).

## Discussion

While we have described three clinical outcomes relevant to opioid use treatment trials, the strength of CTNote:: is its flexibility. The CTNote package and outcomes library will be useful to future substance use disorder clinical trials in the National Drug Abuse Treatment Clinical Trials Network [16], other substance use disorder research, and any other study where the outcome can be well described as a categorical variable with repeated assessments through time. Moreover, representing use patterns as a “word” will allow clinicians and care providers an “at a glance” assessment of participants’ responses to treatment over time and even highlight subgroups of participants by their UDS results.

Additionally, our work adheres to the Open Knowledge [17] philosophy of science, so the code for the CTNote package and all related algorithms are open-source, meaning that anyone who wishes to understand what is going on “under the hood” can study the engine that does the calculations. Further, the library is extensible if anyone wishes to expand our code. These strengths all come without extensive data processing steps in proprietary software: our software now (and all software our team develops with public funds) will remain open source and free to use.

As an aside, we developed this technique with SUD treatment in mind, but we claim that this “word” structure can be applied with minimal modifications to encapsulate and summarize *any* discrete-time categorical stochastic process without information loss. For example, a behavioral intervention could assess participant suicidal ideation or depression each day or week of observation via a five-level Likert scale. A participant’s results over time could then be summarized by a quinary word comprised of the numerals from “1” to “5”. Another example could be a daily assessment of pain during cancer treatments with “none”, “low”, “medium”, and “high” options represented by the letters “N”, “L”, “M”, and “H”. The code base developed in our software package can be directly applied to these examples as well, though the algorithms for detecting specific patterns of behavior should be modified to fit the context of interest.

### Accounting for multivariate categorical variables

These use pattern summaries are substance or substance group specific (*univariate*, statistically speaking). However, a typical urine dipstick tests for up to 12 substances simultaneously. If we were to inspect the participant’s complete urine screening record, we might see an increase in use of some other substances. Specifically, while our example participant decreased their opioid use, their cocaine use may *not* decrease over the same time interval. The **opioid** use pattern summary cannot display information about concurrent **cocaine** use. However, certain patterns of correlations in UDS are immediate: when patients cease treatment and no longer come to treatment visits, all measures of substances become missing, and therefore specifying missing for the entire clinic visit is sufficient. This is an important area of future research: expanding the support of substance use pattern “words” to include poly-substance use in a meaningful way (including the ability to preserve cross-substance correlations) [18].

### The complexity-readability tradeoff

In order to ensure that use pattern summaries work regardless of a computer’s locale or operating system, we limit the symbols in the “word” to proper symbols from the American Standard Code for Information Interchange (ASCII) list [19], which has been a computing standard for decades [20]. Technically speaking, there are 128 7-bit ASCII symbols, but only 96 are visible (printable) characters [21]. Put simply, these 96 characters are all the symbols that a traditional North American computer keyboard can make. Therefore, we could define a substance use pattern “word” with any combination of these 96 printable symbols.

However, such a visual summary with 96 possible values would be incredibly challenging to interpret, consequently failing to meet the requirement that our summary be read easily by a human. In the interest of simplicity, we chose the five symbols mentioned above (+, -, o, *, and _). We recognize that different study designs may necessitate the introduction of additional symbols to this use pattern summary, but we strongly recommend (due to human memory constraints) that such lists be kept to seven symbols or fewer [22]. As an example, we discussed the benefit of adding special symbols for a “missing but excused” clinic visit (or for “urine sample of improper temperature”), but we ultimately did not because such instances were rare in data we had available (three large clinical trials of medication for opioid use disorder containing over 2,500 participants).

### Lossless data summaries

There are no existing methods to create human-readable summaries of TLFB or urine drug screening patterns without losing information. This paper represents a novel application of a decades-old technology used in genetics, engineering, and computer science to solve this problem. Our technique has high fidelity, meaning that hardly any information in the original data is lost when summarized with our use pattern “word”. The one case where information is lost is when multiple assessments for the same substance(s) happen in the same time period for the same participant. We represent such a scenario with the symbol * in our use pattern “words”. In our real-world data sets for medication-assisted treatment of opioid use disorder, this symbol (*) represents only 1.5% of all possible data entries. Therefore, our data summary method unambiguously expresses at least 98.5% of the original clinical trial information without loss.

However, it may be possible to produce a completely lossless compression of the original data, even in these cases of ambiguous UDS results or multiple tests within a single time period. As we mentioned above, there are a total of 96 printable ASCII symbols to choose from, so we could represent up to 96 unique drug test results. For example, we could represent a day with multiple UDS results by a symbol which specifically represents the ordered pattern of results for the substance(s) of interest within that day. In this example, the letter “a” (lower case) could represent two UDS within a single day where the first test was negative for a given substance but the second was positive. Similarly, the letter “A” (upper case) could represent this pattern in reverse order, where the first test was positive and the second was negative. For a computer, such a compression algorithm would be superior to the “quinary word” we propose in this paper (because it would lose less information). However, this compression would be inferior for a human because it would be more challenging to read and interpret. Due to the human memory constraints we mentioned above, we believe that use pattern “words” should be kept to seven symbols or fewer. We believe that metrics of information loss and human processing speed/accuracy could be “traded” by adding more or fewer symbols to our “word”. These metrics could be quantified in future research by cognitive psychologists, but our concern is that—ultimately—the relative value of information loss vs. human processing ability is largely subjective.

### The inflexibility of computers

When discussing how algorithms make decisions about human behavior, we should remember the old maxim of computing: “computers give you what you ask for, not what you want.” The computer cannot understand the many extenuating circumstances that pervade our human experience. Consider a concrete example: assume a participant was supposed to visit the clinic for a weekly urine test on Thursday, but they came on Wednesday instead due to a conflict at their work; their urine sample from Wednesday came back “clean”. What would a clinician at the substance use clinic do in this case? Most people would count the urine screen for that week as negative for the substance(s) of interest.

But what would a computer do? Without the direct input of a human to override the clinical trial protocol, a computer would 1) count the negative urine on Wednesday, and 2) mark the participant as a “failure to appear” / “missing urine screen” on Thursday. Then, because, missing urine screens are imputed as “positive” for the substance of interest in many clinical trial protocols, the computer would 3) write this participant’s urine screen as positive for the Thursday, and then 4) mark the use pattern summary as * (mixed results) instead of - (negative results) for that week, which may be later counted as a use week. While automation is incredibly powerful when done correctly, we must make every effort to ensure that our code “first does no harm”. Further, we must convey this message and provide the underlying code so that users can fully understand what they are getting from its use.

## Conclusion

Here we describe the design decisions and implementation of a code base to summarize behavior through time. We have presented a use case of the quinary word structure to summarize clinical trial participants’ opioid use results as detected by UDS. This example highlights that representing substance use patterns as a “word” allows researchers and clinicians an “at a glance” assessment of participants’ responses to treatment over time. Our machine readable use pattern summaries may serve as a standardized method to calculate treatment outcome and are therefore useful to all future substance use disorder clinical trials. All our work is provided in the open-source R package CTNote. This software, provided under a GPL-3 license, is available free of charge. This affords clinical investigators working with data-scientists/statisticians the opportunity to summarize the patterns of substance use in patients receiving care. While the highest goal of treatment for substance use disorders is abstinence, it is critical to systematically assess other less draconian, harm-reduction endpoints. The functions provided by CTNote give clinical investigators the tools needed to quickly and easily apply any number of definitions of treatment success or failure. We are currently developing such definitions and we welcome collaborations with other research teams that wish to describe response to treatment in any domain.
